# Effect of Calcium Acetate Content on Apatite-Forming Ability and Mechanical Property of PMMA Bone Cement Modified with Quaternary Ammonium

**DOI:** 10.3390/ma13214998

**Published:** 2020-11-06

**Authors:** Haiyang Wang, Toshinari Maeda, Toshiki Miyazaki

**Affiliations:** Graduate School of Life Science and Systems Engineering, Kyushu Institute of Technology, Kitakyushu 808-0196, Japan; wang.haiyang651@mail.kyutech.jp (H.W.); toshi.maeda@life.kyutech.ac.jp (T.M.)

**Keywords:** polymethyl methacrylate (PMMA) bone cement, calcium acetate, apatite formation, bioactivity, simulated body fluid (SBF), setting time, compressive strength

## Abstract

Polymethyl methacrylate (PMMA)-based bone cement is a popular biomaterial used for fixation of artificial joints. A next-generation bone cement having bone-bonding ability, i.e., bioactivity and antibacterial property is desired. We previously revealed that PMMA cement added with 2-(tert-butylamino)ethyl methacrylate, γ-methacryloxypropyltrimethoxysilane and calcium acetate showed in vitro bioactivity and antibacterial activity. This cement contains calcium acetate at 20% of the powder component. Lower content of the calcium acetate is preferable, because the release of a lot of calcium salt may degrade mechanical properties in the body environment. In the present study, we investigate the effects of calcium acetate content on the setting property and mechanical strength of the cement and apatite formation in simulated body fluid (SBF). The setting time increased and the compressive strength decreased with an increase in calcium acetate content. Although the compressive strength decreased after immersion in SBF for 7 d, all the cements still satisfied the requirements of ISO5833. Apatite was formed in SBF within 7 d on the samples where the calcium acetate content was 5% or more. Therefore, it was found that PMMA cement having antibacterial properties and bioactivity can be obtained even if the amount of the calcium acetate is reduced to 5%.

## 1. Introduction

Bone cement consisting of polymethyl methacrylate (PMMA) powder and methyl methacrylate (MMA) liquid is an important bone-repairing material used in orthopedic surgery for fixing artificial joints and curing osteoporosis-related fractures [1,2]. The problem that needs to be solved is the loosening between the cement and the implant, since the cement does not show bone-bonding ability, i.e., bioactivity. In order to provide the PMMA cement with bioactivity, it is often mechanically mixed with bioactive ceramic powders, such as bioactive glass, sintered hydroxyapatite and apatite- and wollastonite-containing glass-ceramic A-W [3,4].

Inspired by the bone-bonding principle of bioactive glass [5], the present authors have reported that PMMA cement was provided with the apatite-forming ability in simulated body fluid (SBF) when it was modified with γ-methacryloxypropyltrimethoxysilane (MPS) and water-soluble calcium salts [6,7]. MPS contributes to the formation of silanol (Si-OH) groups, that is able to induce apatite nucleation, while released calcium ions can accelerate the process. This apatite formation in SBF is known to be required for artificial materials to show bioactivity [8].

**Figure 1:**
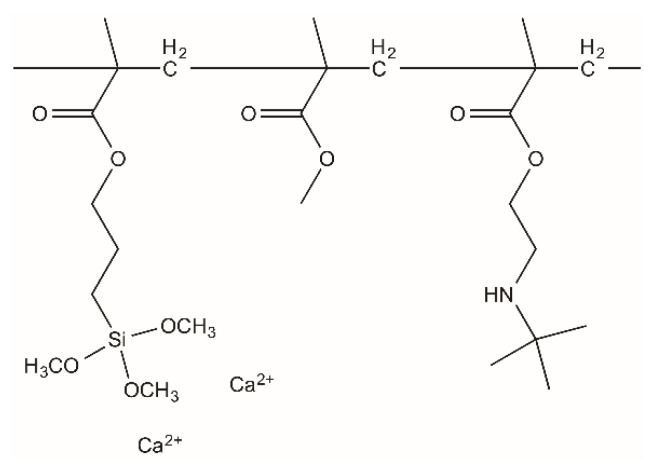
Assumed chemical structure of the cement prepared in this study.

Another challenge faced with PMMA bone cement is bacterial infection when implanted in the human body [9]. The copolymerization of a monomer with an antibacterial quaternary ammonium group has been attracting much attention [9,10,11,12]. The present authors revealed that PMMA cement modified with 2-(tert-butylamino)ethyl methacrylate (TBAEMA, H_2_C = C(CH_3_)CO_2_CH_2_CH_2_NHC(CH_3_)_3_) as well as MPS and calcium acetate (Figure 1) shows apatite formation in SBF and antibacterial activity against both Gram-positive and Gram-negative bacteria [13,14]. In this cement, calcium acetate content is 20% of the powder component. Additionally, minimizing the amount of the calcium salt necessary for the apatite formation is preferable, because the addition of a lot of calcium salt degrades mechanical properties in the body environment [15,16]. In the present study, we prepared PMMA bone cements with different calcium acetate contents and the effects of the calcium acetate on setting behavior, mechanical properties and apatite formation in SBF were investigated.

## 2. Materials and Methods

### 2.1. Materials

PMMA powder (MB-4C) was procured from Sekisui Plastics Co., Ltd. (Tokyo, Japan). Calcium acetate monohydrate, MMA and N,N-dimethyl-*p*-toluidine (NDT) were obtained from FUJIFILM Wako Pure Chemical Corporation (Osaka, Japan). TBAEMA was purchased from Sigma-Aldrich Co. LLC, St. Louis, MO, USA). Benzoyl peroxide (BPO) and the chemical for the preparation of SBF were products of Nacalai Tesque Inc. (Kyoto, Japan). MPS was purchased from Shin-Etsu Chemical Co., Ltd. (Tokyo, Japan). The calcium acetate was priorly dried at 220 °C and BPO was recrystallized in ethanol. The other chemicals were of analytical grade and used without further purification.

### 2.2. Cement Preparation

PMMA-based bone cement consists of two parts: powder source and liquid source. The powder source was composed of PMMA, calcium acetate and BPO. The liquid source consisted of MMA, TBAEMA, MPS and NDT. The detailed composition of each phase is shown in Table 1. The mixing ratio of powder/liquid was 1/0.51 (g/g). The powder component was mixed with a mortar. Temperature and relative humidity during mixing was maintained at 23 ± 2 °C and 50 ± 10%, respectively. The cements with different calcium acetate contents were prepared. The cement with x mass% of the calcium acetate content in the powder component is hereafter denoted as “x%CaAC”.

### 2.3. Setting Time Measurement

A mixed paste of 1.5 g of cement was used to determine the setting time. The size of the bone cement was a 10 mm × 10 mm × 1 mm cuboid. A weight of 300 g was loaded onto the mixed paste with a Vicat needle apparatus (A-004, JAPAN MECC Co., Ltd., Tokyo, Japan) with a cross section of 1 mm^2^. The setting time was defined as the time when a trace of the Vicat needle did not remain on the surface of the cement. These tests were repeated 5 times for each constituent.

### 2.4. Mechanical Properties

Cylindrical samples of 6 mm in diameter and 12 mm in height were utilized for compressive strength measurement. All the specimens, before and after soaking in SBF for 7 d, were subjected to a compressive load with a crosshead speed of 1 mm/min controlled by a universal testing machine (Autograph AG, Shimadzu Co., Kyoto, Japan) until fracture occurred. The compressive strength was calculated by the fracture load and the samples’ cross-sectional area. Five specimens were used for each composition. A t-test was used for significance test.

### 2.5. Bioactivity Test in SBF

Bioactivity of the obtained PMMA bone cement was evaluated in vitro by apatite formation on the surface in SBF (Na^+^ 142.0, K^+^ 5.0, Mg^2+^ 1.5, Ca^2+^ 2.5, Cl^−^ 103.0, HCO_3_^−^ 27.0, HPO_4_^2−^ 1.0 and SO_4_^2−^ 0.5 mM). SBF was prepared by dissolving NaCl, NaHCO_3_, KCl, K_2_HPO_4_·3H_2_O, MgCl_2_·6H_2_O, CaCl_2_ and Na_2_SO_4_ into ultrapure water, one by one. The pH of SBF was adjusted at 7.40 by 1 M HCl and 50 mM of tris(hydroxymethyl)aminomethane. Details of SBF preparation were reported in previous literature [17]. The cements were shaped into rectangular pieces with dimensions of 10 × 10 × 1 mm^3^ and then immersed in polystyrene bottles containing 35 mL of SBF at 36.5 °C. After soaking for a certain period of time, the cements were taken out, rinsed with pure water 3 times and dried in the oven at 65 °C for 24 h.

### 2.6. pH Changes in SBF

Cylindrical samples of 6 mm in diameter and 12 mm in height were prepared for the pH test. The cements were immersed in polystyrene bottles containing 35 mL of SBF at 36.5 °C. After soaking for a certain time interval (1, 3, 5 or 7 d), pH was detected by an ion/pH meter (F-23IIC, Horiba Ltd., Kyoto, Japan).

### 2.7. Characterization

Surfaces of the cements were characterized by thin-film X-ray diffractometer (TF-XRD, MXP3V, MAC Science Ltd., Yokohama, Japan), scanning electron microscope (S-3500N, Hitachi Co., Tokyo, Japan) and Fourier transform infrared attenuated total reflection (FT-IR ATR). In the TF-XRD experiments, the incident beam was fixed at 1° to the surface of each substrate and the scan rate was 0.02°·s^−1^. All the cement specimens were coated with Au-Pd thin film, before SEM observation, by using an ion sputter (E-101, Hitachi Co., Tokyo, Japan). In FT-IR ATR, diamond was used as a prism.

## 3. Results and Discussion

**Figure 2:**
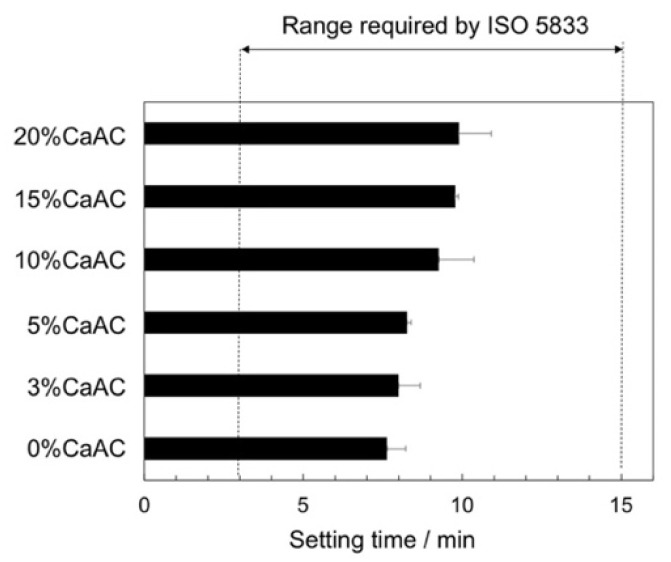
Setting times of the cements mixed with different amounts of calcium acetate (n = 5).

**Figure 3:**
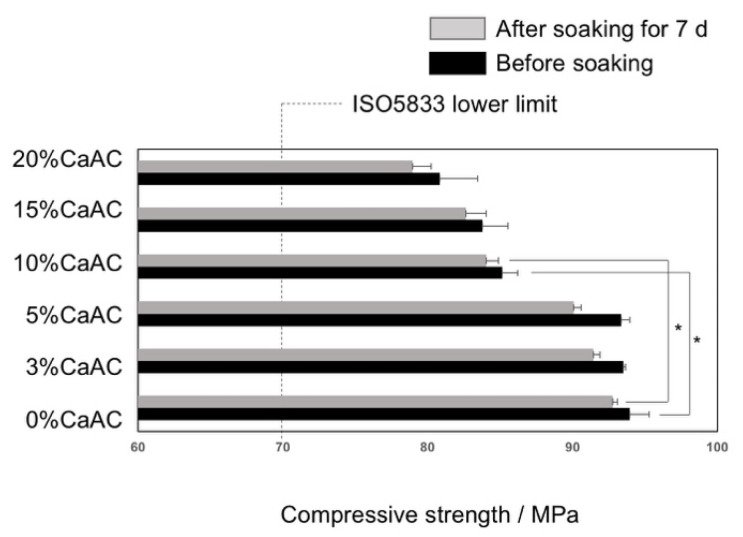
Compressive strength of the cements mixed with different amounts of calcium acetate before and after soaking in SBF for 7 d (n = 5). Asterisk means *p* < 0.05.

Figure 2 shows the setting time of the cements obtained using different amounts of calcium acetate. The setting time increased with an increase in calcium acetate. However, all the cements satisfied the requirements of ISO 5833, irrespective of the calcium acetate content [18]. Figure 3 shows compressive strength of the prepared cements before and after soaking in SBF for 7 d. The compressive strength significantly decreased at a calcium acetate content of 10% or more. It also slightly decreased after soaking in SBF. However, all the cements satisfied the requirements of ISO 5833 even after the soaking (70 MPa at minimum).

An increase in the amount of the calcium acetate extended the setting time. The retard of the setting time by addition of inorganic filler is often observed in bone cement and dental resins [19,20]. This is thought to be because the monomer is adsorbed on the filler surface, its mobility decreases and the viscosity increases to reduce the rate of the radical polymerization [20,21]. Similar phenomenon has occurred in this study. The compressive strength decreased by addition of the calcium acetate. The calcium acetate has a hydrophilic character, but not MMA and PMMA. Therefore, chemical interaction between the calcium acetate and the cement matrix would be low. In addition, grain boundary in aggregation of the calcium acetate would be mechanically weak. It is necessary to increase miscibility with the MMA matrix and improve the dispersibility of the calcium acetate by surface modification in future studies. Additionally, the use of fillers with higher mechanical strength than PMMA would be effective. The decrease in compressive strength in SBF is considered to be due to the release of calcium acetate. However, the following calcium phosphate layer formation in SBF would act as a barrier to suppress the penetration of the surrounding fluid into the cement. Therefore, a significant decrease in the compressive strength would be suppressed.

**Figure 4:**
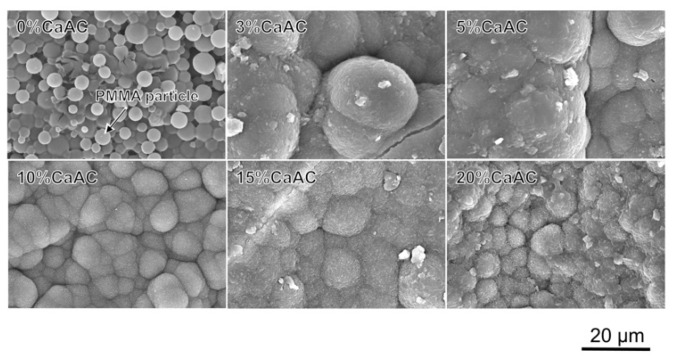
SEM photographs of the surfaces of the cements mixed with different amounts of calcium acetate, which were all soaked in SBF for 7 d.

**Figure 5:**
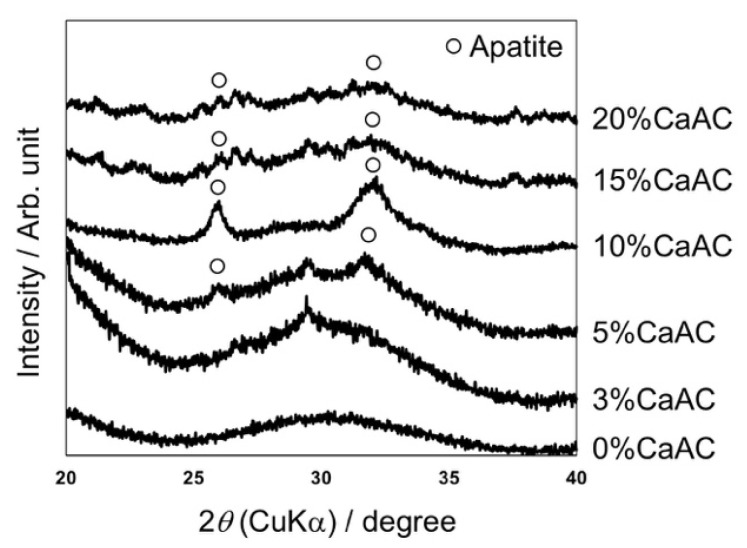
TF-XRD patterns of the surfaces of the cements mixed with different amount of calcium acetate, which were all soaked in SBF for 7 d.

Figure 4 shows SEM photographs of the surfaces of the cements prepared with different amounts of calcium acetate, which were all soaked in SBF for 7 d. Spherical particles of PMMA were clearly observed at 0%, while assemblies of fine particles covered the surfaces of the cements containing calcium acetate. Their morphology was similar to the apatite formed in SBF [22]. TF-XRD patterns of the same samples are shown in Figure 5. Broad peaks assigned to the apatite (JCPDS#09-0432) with low crystallinity were detected around 26° and 32° for the cements containing calcium acetate 5% or more calcium acetate.

**Figure 6:**
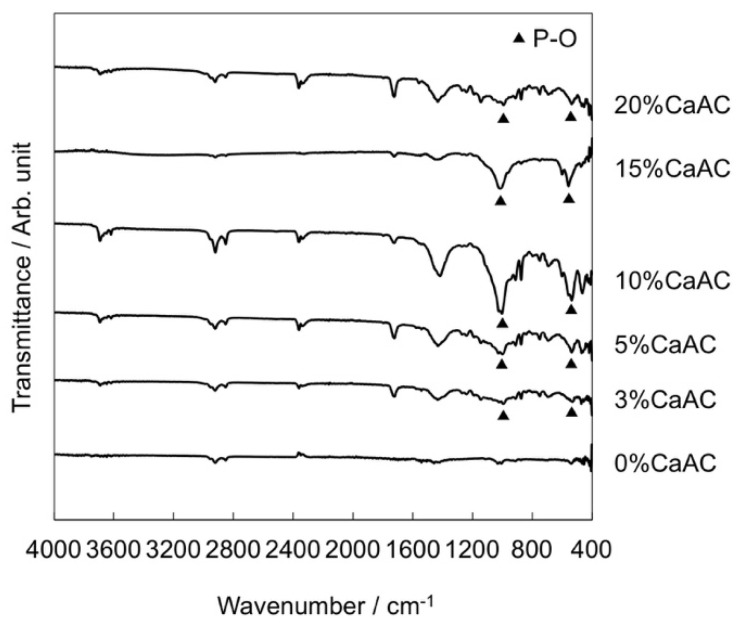
FT-IR ATR spectra of the surfaces of the cements mixed with different amounts of calcium acetate, which were all soaked in SBF for 7 d.

It was found that the addition of the calcium acetate is essential for the apatite formation on the cement in SBF. In the case of the cement with calcium acetate content of 3%, the precipitate was formed similar to the other ones. In addition, peaks assigned to phosphate group were observed in FT-IR ATR spectra around 600 and 1000 cm^−1^ (Figure 6), indicating that the calcium phosphate is formed on the cement surface. Nevertheless, no peak of the apatite was detected by TF-XRD. It is considered that the precipitated calcium phosphate remains in an amorphous state and is not crystallized into the apatite.

A similar phenomenon was observed in poly(hydroxyethylmethacrylate)-based organic-inorganic hybrids added with water-soluble calcium salts [23]. Namely, the hybrids added with the calcium chloride gave diffraction peaks assigned to the apatite, but not those added with the calcium acetate at the same content, in spite of the calcium phosphate layer being formed. Furthermore, Rhee et al. reported that the apatite formation on a collagen film in 1.5 SBF, which has ion concentrations 1.5 times that of SBF, was suppressed when citric acid was added at a high concentration [24]. It has also been reported that Ca^2+^ and CH_3_COO^−^ form a complex (complex formation constant: 0.5 to 1.7) [25]. When a small amount of the calcium acetate is added to the cement, it is assumed that the amount of Ca^2+^ released into SBF is small and the inhibition effect of CH_3_COO^-^ is relatively strong. Therefore, the crystallization of the apatite would be suppressed.

**Figure 7:**
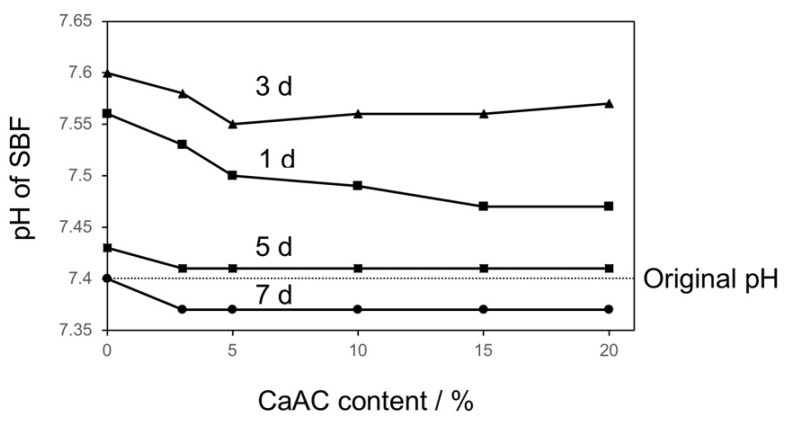
pH variation of SBF after soaking of the cements mixed with different amounts of calcium acetate for different time intervals.

The pH change in SBF after soaking of the cements is shown in Figure 7. It once increased to around 7.55 at initial stage within 3 d followed by a decrease to around 7.37 after 7 d. There was little difference in pH value depending on the calcium acetate content. When the modified cements are soaked in SBF, calcium acetate would mainly be released because MPS and TBAEMA are co-polymerized with MMA. The released calcium acetate increases the pH of the surrounding fluid because of hydrolysis of CH_3_COO^−^ by the following reaction:

(1) $${\mathrm{CH}}_{3}{\mathrm{COO}}^{-}+H_{2}O\to {\mathrm{CH}}_{3}\mathrm{COOH}+{\mathrm{OH}}^{-}$$

Then Si-OH groups formed by hydrolysis of MPS induce the heterogeneous apatite nucleation [5,7]. They grow into a continuous layer because the supersaturation degree of SBF, with respect to the apatite, increases due to the increase in pH and Ca^2+^ concentration. During this process, OH^−^ in SBF is consumed by the following reaction:

(2) $$10{\mathrm{Ca}}^{2+}+6{\mathrm{PO}}_{4}^{3-}+2{\mathrm{OH}}^{-}\to {\mathrm{Ca}}_{10}{\left({\mathrm{PO}}_{4}\right)}_{6}{\left(\mathrm{OH}\right)}_{2}$$

Therefore, a drop of pH in SBF would be subsequently caused.

## 4. Conclusions

This study explored the effect of the calcium acetate on the bioactivity and the mechanical properties of PMMA-based bioactive and antibacterial bone cements. Increasing the amount of the calcium acetate extended the setting time and reduced the compressive strength of the cement, although the measured mechanical properties were in the range of the ISO 5833 requirements. Clear apatite formation was observed at calcium acetate content of 5% or more. Therefore, it was concluded that the addition of calcium acetate at 5% was the most appropriate. On the other hand, the interaction between the calcium salt and MMA monomer and the associated dispersion state effect the setting time and the mechanical strength, so it is necessary to optimize these points in the future.

## Figures and Tables

**Table 1:** Composition of powder and liquid phases of the modified cements.

Powder (per 1 g)/mg			Liquid (per 0.51 g)/mg			
PMMA	BPO	Calcium Acetate	MMA	TBAEMA	MPS	NDT
(971−x)	29	x	486	9.9	9.9	4
